# Event and Comment

**Published:** 1918-04

**Authors:** 


### DENTAL REGISTER

Vol. LXXII APRIL, 1918 No. 4

### EVENT AND COMMENT

**Scientific Prosthetic Restorations**

We are reprinting in this issue a paper read by Dr. Rupert Hall before the Chicago Dental Society, which seems to have created a very interesting,—though a not very conclusive—discussion on the value of measurement of the condyle path in the construction of full dentures. Dr. Hall takes the position that the actual form of the condyle path is not of as much significance as we have been led to suppose it to be, by the elaborate studies made of this matter by our prosthetists. He maintains that the form of the tooth cusps control masticatory efficiency as well as articulation and stability of denture adhesion. This is directly opposed to the ideas upon which practical denture construction has been made during the last ten years. Dr. Hall has devised an articulating frame which differs essentially from all the so-called anatomical articulating frames in that it ignores the usual condyle joints and substitutes a single pivotal point located in the median line and theoretically at the occipital eminence of the skull. This practically takes us back to the plain line or hinge joint articulator, except that it has some adjustment possibilities.

It may take some careful work and more demonstrations to convince most of us of the accuracy of Dr. Hall's methods. There can be no question but that most of our plate prosthesis is so faulty today that were it not for the accommodating character of the condyle joints not one in ten of the full dentures made for patients could be worn at all. For years we have given up trying to teach students to construct full dentures on the basis of exact mathematical formulae. At the same time we have carefully presented the scientific deductions of our researches as a basis for practicable technical methods. The most successful prosthetist we ever knew never used an articulating frame of any kind. He articulated and occluded the teeth directly in the patient's mouth on rigid base plates. He was never a scientist but he was a practical artisan as well as a genius, and an artist. His work satisfied the requirements of his patients. Now we have no first hand knowledge of what Dr. Hall proposes, or how he really does his work, but we can see that he has a practicable scheme that will produce the result which he claims. Whether his means or methods are novel or scientific we can not determine without more intimate acquaintance with them. So far as we can determine they offer possibilities that entitle us to careful examination and practical testing for further confirmation of his claims. We should want to be convinced as to his technical methods in impression and bite making; and we can not quite see how it is practicable to dogmatically define occlusion and articulation in the way he seems to do. Unless harmonious relations are secured, that are individualized in each case, in the placing of the teeth, unnatural restraint is liable to discredit the method because of the unyielding mechanism involved.

**Dentists in the Army**

On March 8, 1918, there were 6,919 dentists registered in the various departments of the army, distributed as follows: In the Dental Corps, 211. In the Reserve Dental Corps, 5,114. In active service, 1,334. National Guard, 260. Allowing one dentist to every one thousand men it would seem that there is already enlisted enough dentists to supply the needs of the army for some time, unless the rate of recruiting is materially increased. It is probable that a considerable number of dental reservists will be called to assist in the medical hospitals, as a considerable shortage of surgeons exists, and dental reservists with surgical training can be used to great advantage as assistants to the medical surgeons. It seems too bad that Congress could not be made to see that more than one dentist to a thousand soldiers would be needed. No dentist can take proper care of a thousand men. At any rate the dentists when sent over will be most useful assistants if needed in hospital work.

**Verdict in the Taggart Suit**

We have received a notice from the President of the Dentists Mutual Protective Alliance, that the alliance has won its suit against Taggart. No particulars are made known and we have no information as to the significance of the verdict. Full details are promised later on for publication.

